# Impact of renal recovery on in-hospital and post-discharge mortality

**DOI:** 10.1590/1980-220X-REEUSP-2023-0144en

**Published:** 2023-12-04

**Authors:** Alberto Augusto Martins Paiva, Marcia Cristina da Silva Magro, Paulo Percio Mota Magro, Tayse Tamara da Paixão Duarte

**Affiliations:** 1 Universidade de Brasília, Faculdade de Ceilândia, Brasília, DF, Brazil.; 2 Instituto Federal de Brasília, Brasília, DF, Brazil.

**Keywords:** Acute Kidney Injury, Recovery of Function, Mortality, Lesión Renal Aguda, Recuperación de la Función, Mortalidad, Injúria Renal Aguda, Recuperação de Função Fisiológica, Mortalidade

## Abstract

**Objective:**

To verify the impact of renal recovery on mortality in non-critically ill patients with acute kidney injury.

**Method:**

A prospective cohort study was carried out in a public hospital in the Federal District with patients with acute kidney injury admitted to a non-critical care unit. Renal recovery was assessed based on the ratio of serum creatinine to baseline creatinine and the patient was followed up for 6 months. Mortality was assessed during hospitalization and after discharge.

**Results:**

Of the 90 patients with hospital-acquired kidney injury, renal recovery was identified in 34.1% to 75% of cases, depending on the time of assessment, considering a follow-up period of up to 6 months. Recovery of renal function during follow-up had an impact on in-hospital mortality [95% CI 0.15 (0.003 – 0.73; p = 0019).

**Conclusion:**

Recovery of renal function has been shown to be a protective factor for mortality in patients admitted to the non-critical care unit. Early identification of kidney damage and monitoring of physiological and laboratory variables proved to be fundamental in identifying the severity of the disease and reducing mortality.

## RESUMO

**Objetivo:**

Verificar o impacto da recuperação renal na mortalidade de pacientes não críticos com injúria renal aguda.

**Método:**

Coorte prospectiva, realizado em um hospital público do Distrito Federal com pacientes diagnosticados com injúria renal aguda internados em uma unidade de cuidados não críticos. A recuperação renal foi avaliada a partir da razão da creatinina sérica em relação à creatinina basal e o paciente foi acompanhado por 6 meses. A mortalidade foi avaliada durante internação e após alta hospitalar.

**Resultados:**

Dos 90 pacientes com injúria renal adquirida no hospital, identificou-se a recuperação renal em 34,1% a 75% dos casos, a depender do momento de avaliação, considerando o período de acompanhamento de até 6 meses. A recuperação da função renal durante o acompanhamento impactou na mortalidade intra-hospitalar [IC 95% 0,15 (0,003–0,73; p = 0019).

**Conclusão:**

A recuperação da função renal demonstrou-se como um fator protetor para mortalidade em pacientes internados na unidade de cuidados não críticos. A identificação precoce da injúria renal e o monitoramento de variáveis fisiológicas e laboratoriais mostraram-se fundamentais para identificação da gravidade da doença e redução da mortalidade.

## RESUMEN

**Objetivo:**

Verificar el impacto de la recuperación renal en la mortalidad en pacientes no críticos con lesión renal aguda.

**Método:**

Cohorte prospectiva, realizada en un hospital público del Distrito Federal con pacientes con daño renal agudo ingresados en una unidad de cuidados no críticos. La recuperación renal se evaluó según la relación entre la creatinina sérica y la creatinina inicial y se siguió al paciente durante 6 meses. La mortalidad se evaluó durante la hospitalización y después del alta hospitalaria.

**Resultados:**

De los 90 pacientes con daño renal intrahospitalario, se identificó recuperación renal entre el 34,1% y el 75% de los casos, dependiendo del momento de la evaluación, considerando un período de seguimiento de hasta 6 meses. La recuperación de la función renal durante el seguimiento tuvo impacto en la mortalidad hospitalaria [IC 95% 0,15 (0,003 – 0,73; p = 0019).

**Conclusión:**

La recuperación de la función renal demostró ser un factor protector de la mortalidad en pacientes ingresados en la unidad de cuidados no críticos. La identificación temprana de la lesión renal y el seguimiento de variables fisiológicas y de laboratorio resultaron esenciales para identificar la gravedad de la enfermedad y reducir la mortalidad.

## INTRODUCTION

Acute kidney injury (AKI), despite growing scientific knowledge, results in a risk of short- and long-term mortality and causes high costs for public health systems^(1)^. It is a clinical condition characterized by an abrupt reduction in the kidneys’ ability to adequately filter the blood and triggers the accumulation of toxic metabolic products and hydroelectrolytic disturbances^(2)^.

AKI is a frequent complication of hospitalization and is independently associated with progressive deterioration of kidney function, which can progress to chronic kidney disease (CKD), including end-stage renal disease^(3)^. Hospital-acquired AKI (HA-AKI) results from a sustained increase in serum creatinine (sCr) of 0.3 mg/dL compared to baseline and/or a decrease in urine output (UO) of 0.5 ml/kg/h after 48 hours of hospitalization^(4,5)^.

In non-critical care units, such as wards, AKI has a lower incidence when compared to the intensive care environment, but it still has an impact on long-term adverse outcomes, a condition that is highly dependent on the presence of pre-existing comorbidities. Regardless of the origin of AKI, early identification of at-risk patients is essential to avoid progression to an advanced and irreversible condition^(6)^. Therefore, identifying risk factors facilitates the adoption of appropriate preventive and therapeutic measures to avoid or delay the progression of renal impairment^(7)^, considering that there is no successful pharmacological therapy for the treatment of AKI, which maintains renal replacement therapy (RRT) as an essential component in the treatment of this pathology^(6)^.

Although some risk factors for AKI are not modifiable, for example advanced age or pre-existing CKD, others are preventable or potentially modifiable, for example exposure to nephrotoxic drugs. A better understanding of the risk of acquired AKI and the identification of potentially modifiable risk factors are essential to reduce the occurrence of this syndrome^(8)^.

Measures that contribute to recovering renal function and reducing mortality in hospitalized patients generally include early identification, monitoring of renal function biomarkers and adjustment of nephrotoxic drug doses. Recovery of kidney function is crucial for a better patient prognosis, especially in a hospital setting^(4,5)^. In many cases, kidney recovery is possible, especially if AKI is diagnosed and treated early. However, in some severe cases, resources may be limited, resulting in a higher mortality rate^(9)^.

In view of the above, the aim of this study was to verify the impact of renal recovery on mortality in non-critically ill patients with HA-AKI.

## METHOD

### Type of Study

This is a prospective cohort, guided by the recommendations of the Strengthening the Reporting of Observational Studies Epidemiology (STROBE)^(10)^.

### Population, Location, Selection Criteria

A total of 1,250 patients admitted to the medical ward of a tertiary public hospital located in the western region of the Federal District (Brazil), part of the Brazilian Health System, known as SUS (*Sistema Único de Saúde*), were evaluated.

Patients were included if they were over 18 years old, had been hospitalized in the ward for more than 48 hours and had a sustained change in serum creatinine ≥ 0.3 mg/dL compared to baseline for at least 48 hours^(8)^. Patients with a glomerular filtration rate (GFR) <30mL/min/1.73m^2^, on maintenance dialysis and/or undergoing emergency surgery after identification of HA-AKI were excluded.

### Population, Location, Selection Criteria

**Figure 1:**
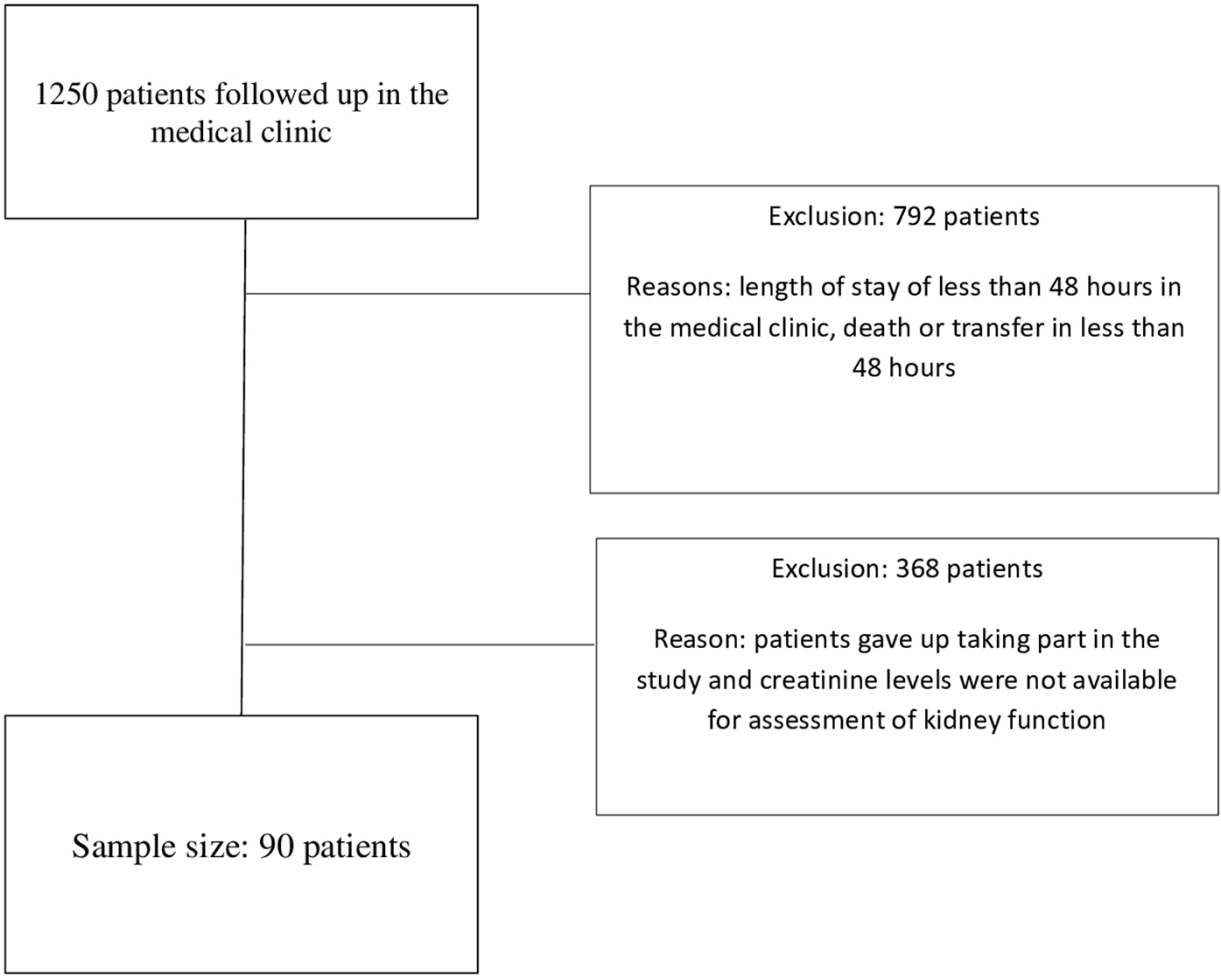
Flowchart of the research sample size.

Out of a total of 1,250 adult patients admitted to the medical ward, a follow-up of up to six months was carried out on 90 patients who developed HA-AKI according to the creatinine criterion of the Kidney Disease: Improving Global Outcomes (KDIGO) classification^(2)^. Sample losses were due to hospitalization time of less than 48 hours in the medical clinic, death or transfer in less than 48 hours (n = 792), patients giving up on participating in the study and absence of creatinine dosage to assess renal function (n = 368) (Figure 1).

The sample size calculation considered a power of 80% and was obtained using the formula^(11)^:

2[zα
2(pq¯
)12+zβ
(p1q1+p2q2)12]2(1+(n−
1)ρ
)n(p1−
p2)2

Where: p1 is the proportion of individuals who had complete recovery from renal dysfunction at the first level of the categorical variable; p2 is the proportion of individuals who had complete recovery from renal dysfunction at the second level of the categorical variable; q1 = 1 – p1; q2 = 1 – p2; $\bar{\text{p}}$ = (p1 + p2) / 2; $\bar{\text{q}}$ = 1 – $\bar{\text{p}}$ ; ρ is the intra-class correlation; n is the number of measurements on the same individual; z_α_ is the percentile of the normal distribution corresponding to the significance level; z_β_ is the percentile of the normal distribution corresponding to the power of the test.

The final sample consisted of 90 patients with HA-AKI during their hospital stay in the medical ward. The cohorts were divided into patients who died during their stay in the ward and those who died after hospital discharge.

We classified the severity of HA-AKI according to the KDIGO classification, as follows: (Stage 1) risk of injury characterized by a 1.5 to 1.9-fold increase in baseline creatinine; (Stage 2) kidney injury expressed by a 2.0 to 2.9-fold increase in baseline creatinine, or (Stage 3) kidney failure characterized by a 3.0-fold increase in baseline creatinine or a 4.0 mg/dL increase or the start of dialysis therapy^(2)^.

Recovery of renal function was calculated in patients with HA-AKI by means of the serum creatinine ratio (sCr) in relation to baseline sCr, and they were then classified as: (1) Full recovery of renal function: creatinine returns to baseline sCr; (2) No recovery: sCr remains above 1.5x baseline^(12)^, at 4 points in time, namely: 10 days after the HA-AKI was identified, 30 days after the HA-AKI was identified, at the time of hospital discharge and 6 months after the HA-AKI.

Baseline creatinine was based on three strategies: (1) 7 to 365 days before hospital admission; (2) Lowest Cr value in the first 7 days of hospital admission; or (3) Hospital admission^(13)^. The reference value for serum creatinine used in this study was 0.7 to 1.2 mg/dL, which reflects the normal level of kidney function adopted by the Federal District Health Department.

The reference values adopted by the Federal District Health Department for laboratory tests were: urea between 8 and 20 mg/dL, sodium between 136 and 145 mEq/L, potassium between 3.5 and 4.5 mEq/L and leukocytes between 4,000 and 11,000 μL. Overweight individuals had a body mass index (BMI) between 25 and 29.9 kg/m^2^ and obese individuals had a BMI ≥ 30kg/m^2(14)^. Normothermia, temperature between 36°C and 37.2°C, febrile state between 37.3°C and 37.7°C and hyperthermia > 37.8^(15)^.

### Data Collection

Data collection took place between January 2020 and December 2021. The researchers prepared a structured questionnaire consisting of objective items related to clinical and demographic variables, such as gender (male and female), age, weight, height, body mass index, hospital outcome (death; discharge; referral to another unit), post-discharge deaths, ethnicity (nothing to declare; white; black; brown), whether they had been admitted to another unit before the medical clinic, blood transfusion and use of non-steroidal anti-inflammatory drugs (NSAIDs) and angiotensin-converting enzyme inhibitors (ACEIs) before admission to the medical clinic, renal replacement therapy (hemodialysis) in the non-critical unit, mobility (bedridden; ambulant), level of consciousness (conscious; torporous; comatose; confused; absent), comorbidities, blood transfusion during hospitalization, type of oxygen therapy, medications used, laboratory tests related to renal function (creatinine, urea, creatinine clearance), electrolytes (sodium, potassium), hematocrit, leukocytes, hemodynamic variables and axillary temperature.

Data collection procedures

**Stage I:** Weekly assessment of clinical and laboratory records in the patient’s electronic medical record. Patients were included when there was a sustained increase in serum creatinine for at least 48 hours, according to the KDIGO guidelines for HA-AKI^(2)^.

**Stage II:** Following the identification of HA-AKI, blood samples were taken daily by the medical ward staff to monitor the patients’ kidney function, according to the pre-established protocol and the patient’s clinical stability.

**Stage III:** The laboratory parameters were monitored daily for 15 days and then in months one, two, three and six after the HA-AKI was identified, by consulting the electronic medical record regardless of whether the patient was hospitalized or not.

**Stage IV:** During the period of hospital discharge, a system of face-to-face visits was set up by the researchers with the aim of providing requests for laboratory tests and guidance on the laboratory collection of serum creatinine to assess renal function. The laboratory collection was carried out at the Primary Care Unit (PCU) closest to the patient’s home, in the post-discharge period, by PCU staff.

**Stage V:** During the post-discharge follow-up period, which lasted six months, the researchers adopted a telemonitoring strategy through telephone contact, with the aim of reinforcing the guidelines on collecting serum creatinine at the PCU. If there were any changes in the results of the laboratory tests, the patient was referred for a medical or nursing consultation at the nearest PCU to their home.

### Data Analysis

Descriptive analysis was carried out by calculating summary and dispersion measures. The hypothesis of independence between variables was tested using Pearson’s chi-square and Fisher’s exact tests. The hypothesis of adherence of continuous variables to normal distribution was tested using the Shapiro-Wilks test and as this was rejected in all the tests carried out, the Mann-Whitney test was used to assess the hypothesis of equality of medians. Crude and multivariate models were built using simple and multivariate logistic regression for KDIGO, in-hospital death and death after discharge. To this end, the following inclusion criteria were established for the models: i) significant variable at the 0.1 level in the univariate hypothesis tests; ii) absence of separation phenomena; iii) number of absentees not exceeding 10% individually and 15% globally. Crude and multivariate odds ratios and their respective confidence intervals were estimated. The significance level adopted in the study was 5% and the software used was R Core Team 2022 (version 4.2.1).

### Ethical Aspects

The study was approved by the Research Ethics Committee of the Foundation for Teaching and Research in Health Sciences of the Health Department of the Federal District, in accordance with Resolution 466/2012, according to Opinion No. 3.327.399, approved on May 15, 2019. All the patients who took part in the study signed an informed consent form.

## RESULTS

Of the total of 90 patients with HA-AKI included in the study, the majority were male (55.6%), of brown ethnicity (81.1%) and aged 62.54 (±15.5) years (Table 1).

**Table 1:** Clinical and demographic characteristics of patients with acute kidney injury (n = 90). Brasília, DF, Brazil, 2020/2021.

Variables	n (%)	Mean (±SD)	Median (25–75)
**Sex**			
Male	50 (55.6)	–	–
Female	40 (44.4)	–	–
**Age (anos)**	–	62.54 (15.5)	64.5 (50–74)
**Weight (kg)**	–	70.83 (16.19)	68 (61.75–79.5)
**Height (cm)**	–	162.76 (10.48)	161 (155.5–168)
**BMI (kg/m** ^2^ **)**	–	26.46 (4.7)	26.49 (23.12–29.81)
**COVID**	21 (23.3)	–	–
**Outcome**			
Mortality	13 (14.4)	–	–
Discharge	55 (61.1)	–	–
Referral to another unit	22 (24.4)	–	–
Post-discharge deaths	22 (28.6)	–	–
**Ethnicity**			
Nothing to declare	1 (1.1)	–	–
White	14 (15.6)	–	–
Black	2 (2.2)	–	–
Brown	73 (81.1)	–	–
**Clinical data of patients pre-admission to the medical clinic**			
Admitted to another unit	81 (90.0)	–	–
Received blood transfusion	17 (21.0)	–	–
Used antibiotics	42 (51.9)	–	–
Use of NSAIDs	27 (33.3)	–	–
Use of ACE inhibitors	24 (29.6)	–	–
**Mobility during hospitalization**			
Bedridden	29 (32.2)	–	–
Wandering	61 (67.8)	–	–
**Level of consciousness** ^1^			
Awareness	73 (81.1)	–	–
Torporous	5 (5.6)	–	–
Comatose	4 (4.4)	–	–
Confused	7 (7.8)	–	–
**Comorbidities**			–
Diabetes mellitus	48 (53.3)	–	–
Systemic arterial hypertension	65 (72.2)	–	–
Respiratory diseases	25 (27.8)	–	–
Heart disease	50 (55.6)	–	–
Hepatopathy	10 (11.1)	–	–
Neoplasms	9 (10.0)	–	–
Smoking	25 (27.8)	–	–
Alcoholism	22 (24.4)	–	–
**Blood transfusion**	8 (8.9)	–	–
**Axillary temperature (°C)**	–	36.37 (0.7)	36.3 (36.03–36.6)
**Oxygen therapy**			
Nasal cannula	13 (14.4)	–	–
Oxygen mask	9 (10.0)	–	–
Non-invasive ventilation	2 (2.2)	–	–
Tracheostomy (macronebulization)	12 (13.3)	–	–
**KDIGO**			
1	31 (34.4)	–	–
2	27 (30.0)	–	–
3	32 (35.6)	–	–
**Kidney recovery**			
10 days	14 (34.1)	–	–
30 days	23 (46.9)	–	–
Medical discharge	23 (45.1)	–	–
6 months	6 (75.0)		–
**HD dependency after discharge**	12 (14.6)	–	–

N – Absolute frequency; SD – Standard deviation; BMI – Body Mass Index; NSAID – Non-steroidal anti-inflammatory drugs; ACEI – Angiotensin-converting enzyme inhibitor; KDIGO – Kidney Disease: Improving Global Outcomes; °C – Degrees Celsius; HD – hemodialysis; 1 – Data missing in 1 patient.

Most of the patients (90%) who had been hospitalized in other units prior to admission to the medical clinic ward required blood transfusions (21%), antibiotics (51.9%) and non-steroidal anti-inflammatory drugs (33.3%). During their stay in the medical ward, we observed that the patients were bedridden (32.2%), although conscious (81.1%) and clinically normothermic (36.3°C). The most frequent comorbidities were systemic arterial hypertension (72.2%), heart disease (55.6%) and diabetes mellitus (53.3%) (Table 1).

When evaluating HA-AKI, the majority of patients had low and intermediate severity levels, KDIGO 1 and 2 (64.4%), to the detriment of those with KDIGO 3 (35.6%). The occurrence of recovery of renal function varied according to time. Shorter periods resulted in a lower percentage of renal recovery, 34.1% (10 days) and in the long term renal recovery was more frequent, 75.5% after 6 months (Table 1).

Hospital discharge was the most frequent clinical outcome (61.1%) compared to death during hospitalization (14.4%), a condition described in Table 1.

A history of hospitalization in other pre-medical clinic hospital units (p = 0.023) and the need for supplementary in-hospital oxygen therapy (p = 0.038) were associated with the occurrence of death. Metabolic changes in sodium (145.29 ± 11.17 mEq/L; p = 0.01), creatinine at the time of hospital discharge (2.64 ± 1.27 mg/dL; p = 0.006), potassium (3.83 ± 0.75 mEq/L; p = 0.007), leukocytes (15.47 ± 10.01 mm^3^; p = 0.009) and axillary temperature (36.88 ± 0.8 °C; p = 0.005) showed a correlation with in-hospital death (Table 2).

**Table 2:** Association of clinical, demographic, hemodynamic and laboratory variables with the outcome of death during (n = 13) and after hospital discharge (n = 22). Brasília, DF, Brazil, 2020/2021.

Variables	In-hospital death (n = 13)				Post-discharge death (n = 22)			
	n (%)	Mean (SD)	Median (27–75)	p-value	n (%)	Mean (SD)	Median (25–75)	p-value
**Sex**								
Male	9 (69.2)	–	–	0.371	11 (50)	–	–	0.625
Female	4 (30.8)	–	–		11 (50)			
**Age**	–	62.62 (13.49)	64 (61–72)	0.931	–	60.73 (15.38)	62.5 (49–73)	0.542
**Height**	–	165.67 (9.23)	165.5 (159–170)	0.196	–	160.48 (9.76)	160 (150–167)	0.292
**BMI**	–	24.77 (3.65)	24.59 (23.25–26.46)	0.143	–	25 (4.15)	26.56 (21.5–28.58)	0.142
**COVID**	1 (7.7)	–	–	0.285	7 (31.8)	–	–	0.384
**Ethnicity**								
Nothing to declare	0 (0)	–	–		1 (4.5)	–	–	0.327
White	2 (15.4)	–	–	1.000	4 (18.2)	–	–	
Black	0 (0)	–	–		0 (0)	–	–	
Brown	11 (84.6)	–	–		17 (77.3)	–	–	
**Pre-admission to another unit in the medical clinic**	9 (69.2)	–	–	0.023	22 (100)	–	–	0.106
**Use of antibiotics in the medical clinic**	5 (55.6)	–	–	1.000	10 (45.5)	–	–	0.618
**Pre-admission to the medical clinic**								
**Use of NSAIDs**	3 (33.3)	–	–	1.000	4 (18.2)	–	–	0.112
**Use of ACE inhibitors**	4 (44.4)	–	–	0.439	2 (9.1)	–	–	0.014
**Use of beta-lactam antibiotics**	10 (76.9)	–	–	0.537	19 (86.4)	–	–	0.037
**Dependence on hemodialysis after discharge from the medical ward**	1 (11.1)	–	–	1.000	7 (31.8)	–	–	0.013
**Comorbidities**								
Diabetes Mellitus	5 (38.5)	–	–	0.368	11 (50)	–	–	0.808
Systemic Arterial Hypertension	10 (76.9)	–	–	1.000	16 (72.7)	–	–	1.000
Respiratory Diseases	3 (23.1)	–	–	1.000	7 (31.8)	–	–	0.785
Heart Disease	4 (30.8)	–	–	0.071	14 (63.6)	–	–	0.463
Hepatopathy	3 (23.1)	–	–	0.155	4 (18.2)	–	–	0.251
Neoplasms	1 (7.7)	–	–	1.000	4 (18.2)	–	–	0.214
**Blood transfusion in the medical clinic**	0 (0)	–	–	0.597	5 (22.7)	–	–	0.019
**Oxygen therapy**								
Ambient air	4 (30.8)	–	–	0.038	11 (50)	–	–	
Nasal cannula	2 (15.4)	–	–		4 (18.2)	–	–	0.490
Oxygen mask	4 (30.8)	–	–		4 (18.2)	–	–	
Non-invasive ventilation	1 (7.7)	–	–		0 (0)	–	–	
Tracheostomy (macronebulization)	2 (15.4)	–	–		3 (13.6)	–	–	
**KDIGO**								
1	3 (23.1)	–	–	0.530	7 (31.8)	–	–	1.000
2-3	10 (76.9)	–	–		15 (68.2)	–	–	
**Laboratory parameters**								
Creatinine (mg/dL)	–	2.17 (0.8)	2.03 (1.74–2.39)	0.124	–	2.53 (1.94)	2.23 (1.55–2.96)	0.065
Urea (mg/dL)	–	102.32 (43.57)	103.06 (55.25–119.37)	0.253	–	111.67 (57.96)	117.24 (60.68–136.7)	0.020
Sodium (mEq/L)	–	145.29 (11.17)	147.65 (136.44–151.41)	0.010	–	138.01 (11.62)	134.89 (129.68–140.73)	0.615
Potassium (mEq/L)	–	3.83 (0.75)	3.93 (3.13–4.14)	0.007	–	4.55 (0.89)	4.33 (4.02–5)	0.567
High Creatinine	–	2.64 (1.27)	2.34 (1.8–3.49)	0.006	–	2.39 (2.07)	1.8 (1.1–3.23)	0.244
Leukocytes (μL)	–	15.47 (10.01)	13.26 (10.3–15.72)	0.009	–	21.93 (47.36)	10.34 (8.12–13.01)	0.645
**Axillary temperature (°C)**	–	36.88 (0.8)	36.67 (36.45–37.25)	0.005	–	36.37 (0.68)	36.2 (35.93–36.54)	0.338

N – Absolute frequency; SD – Standard deviation; BMI – Body Mass Index; NSAID – Non-steroidal anti-inflammatory drugs; ACEI – Angiotensin-converting enzyme inhibitor; CM – Clinical Medicine; Cr – Creatinine; KDIGO – Kidney Disease: Improving Global Outcomes.

Mortality after hospital discharge was significant among patients who required angiotensin-converting enzyme inhibitors (ACEIs) in other sectors prior to admission to the medical ward (p = 0.014), beta-lactam antibiotics during their stay in the medical ward (p = 0.037), and had high urea levels (111.67 ± 57.96 mg/dL; p = 0.020). There was also a correlation between post-discharge mortality and the need for blood transfusion during a stay in the medical ward (n = 5; 22.7%; p = 0.019) and dependence on hemodialysis (n = 7; 31.8%; p = 0.013) (Table 2).

Patients with hyperthermia during their stay in the medical clinic ward were 3.72 times more likely to die in hospital (95% CI 1.39–10.00; p = 0.009) (Table 3).

**Table 3:** Multivariate analysis of the association between variables and death during and after hospital discharge. Brasília, DF, Brazil, 2020/2021.

Variables	In-hospital death		Post-discharge death	
	ORm (95%CI)	p-value	ORm (95%CI)	p-value
Axillary temperature (°C)	3.72 (1.39-10.00)	0.009	–	–
Dependence on hemodialysis after discharge from the Medical Clinic	–	–	11.19 (2.17–57.84)	0.004
Blood transfusion in the Medical Clinic	–	–	19.28 (3.44–108.13)	0.001

ORm – Multivariate odds ratio; °C – Degrees Celsius.

We found that those dependent on hemodialysis after discharge from the medical ward (ORm 11.19; 95% CI 2.17–57.84; p = 0.004) and the need for blood transfusion during hospitalization (95% CI 3.44–108.13; p = 0.001) were, respectively, 11.19 and 19.28 times more likely to die after hospital discharge (Table 3).

There was a correlation between renal recovery and in-hospital mortality (OR 0.15; 95% CI 0.03 – 0.73; p = 0.019). Renal recovery was shown to be a protective factor for death in patients who developed HA (Table 4).

**Table 4:** Association between short- and long-term renal function recovery and mortality among patients with hospital-acquired acute kidney injury. Brasília. DF, Brazil, 2020/2021.

	In-hospital death		Post-discharge death	
	OR (95%CI)	p-value	OR (95%CI)	p-value
**Recovery**				
Discharge	–	–	0.27 (0.06–1.14)	0.074
10 days	0.27 (0.03–2.50)	0.248	0.40 (0.07–2.19)	0.288
30 days	0.19 (0.02–1.77)	0.145	1.84 (0.49–6.87)	0.366
Total	0.15 (0.03–0.73)	0.019	0.83 (0.32–2.19)	0.711

OR – Odds Ratio. 95%CI – 95% confidence interval.

## DISCUSSION

The present study confirmed that recovery of renal function was shown to be a protective factor against mortality, especially in overweight and elderly male patients who develop HA-AKI during hospitalization in a medical ward. Understanding the timing of recovery, as seen in the current evidence, helps to identify different phenotypes of HA-AKI prone to adverse outcomes^(16)^.

Renal recovery in cardiac surgery is estimated at 44 to 84% of patients^(16)^. In our findings, in a non-critical care environment, such as the ward, renal recovery showed a progressive trend and reached 75% of patients over the 180-day follow-up. Late recovery of renal function confers a greater risk of chronic critical illness with poor survival and compromised quality of life in the long term(17), This is consistent with our results, which showed the need for post-discharge hemodialysis in 14.6% of patients and gave patients an 11.19 chance of death after hospital discharge.

In this sense, recovery of renal function is essential in reversing physiological alterations, as well as mitigating the need for dialysis or kidney transplantation, which improves water and electrolyte balance and reduces the inflammatory burden, as well as preventing additional complications, which has been fundamental in improving patient prognosis and reducing mortality^(18)^.

Failure to recognize AKI at an early stage by monitoring renal function results in the development of HA-AKI and potential progression to CKD^(19)^. HA-AKI, KDIGO 1 and 2 (mild and moderate severity) was identified more frequently in the medical ward (64.4%) than in the more severe ward (KDIGO 3). The stage of severity can be interpreted by the magnitude of the structural and functional derangement of the organ and contributes to quantifying renal recovery^(20)^, which, in our study and overall, was more frequent during hospitalization in the ward. The clinical trajectory and renal recovery among hospitalized patients can be used to optimize prevention and early diagnosis^(16)^. A condition that endorses the relevance of monitoring physiological and laboratory variables, also evidenced in our findings, as a measure to mitigate the severity of HA-AKI and consequently patient mortality.

Early identification of HA-AKI has been hampered by a wide variety of clinical conditions common in developed and developing countries, such as Brazil(21) such as body temperature, the need for blood transfusion and dependence on hemodialysis after discharge, which in a multivariate analysis of our findings showed a correlation with mortality during hospitalization and after discharge. Variables such as body temperature, when reduced, for example, in-hospital, can even result in physiological changes such as increased vascular resistance and decreased oxygen consumption, with impaired renal function^(22)^.

The transfusion of red blood cells described represents a modifiable risk factor that can induce oxidative stress and systemic inflammatory response syndrome with a greater risk of death, due to deterioration in oxygen supply and vascular regulation, thus damaging the kidneys. Therefore, maintaining normovolemia, reducing blood loss and avoiding unnecessary blood transfusions are extremely important to prevent AKI from occurring^(23)^. Systematic review describes that the high severity of HA-AKI is associated with increased mortality from different causes^(24)^ as shown in our study, where even patients with more severe HA-AKI (KDIGO 2 and 3) had a greater tendency to mortality both in-hospital and post-discharge, although this was not significant, and patients with persistent need for hemodialysis^(25)^ after hospital discharge, as evidenced in our study, showed a significant risk of mortality. In this way, identifying patients at risk can play an important role in supporting healthcare professionals, since around 11% of deaths in the hospital setting occur due to a failure to promptly recognize and treat patients in clinical deterioration(26).

The etiology of HA-AKI is heterogeneous, and its impact on short- and long-term outcomes (in-hospital and post-discharge) depends on residual renal function, the capacity for repair after renal stress and different factors which, through different pathophysiological pathways, can generate an imbalance in oxygen supply and demand and result in hypoxemia and oxidative stress, which result in endothelial damage, activation and inflammation of the immune system, interstitial edema and vasoconstriction, which in turn further decrease oxygen supply^(27)^ such as anemia expressed by the need for blood transfusion and hypoxemia.

The use of ACE inhibitors can increase the risk of hyperkalemia, hypotension and the need for RRT, contributing to negative outcomes^(28)^. Just as the use of antibiotics can induce renal toxicity due to nephrotoxicity^(29)^ and induce HA-AKI. These therapeutic strategies, described in the current investigation, can trigger damage to the renal structure over time and have an impact on the risk of death, with dependence on hemodialysis, body temperature and the need for blood transfusion being independent risk factors for mortality^(30)^.

The results of our study indicate that recovery analyzed at different times, including after hospital discharge, when present, can improve prognosis by protecting against mortality. In addition, knowing the factors that influence short- and long-term mortality in a non-critical care setting can contribute to properly targeting strategies and improving patients’ quality of life.

The main limitations of this study are related to its single-center nature, the small sample size and the difficulty patients had in adhering to laboratory tests after hospital discharge. The risk of measurement bias is highlighted by the collection of hemodynamic variables in the patient’s electronic medical record, as well as the lack of variables at different times and the absence of associations between urine output and other clinical variables due to the scarcity or inaccuracy of their recording in the ward.

## CONCLUSION

Recovery of renal function is an important protective factor for mortality in patients with HA-AKI hospitalized in a non-critical care unit. Early identification of HA-AKI and monitoring of physiological and laboratory variables proved to be fundamental in identifying the severity of the disease and reducing mortality.

## References

[1] Halmy L, Riedel J, Zeman F, Tege B, Linder V, Gnewuch C (2021). Renal recovery after the implementation of an electronic alert and biomarker-guided kidney-protection strategy following major surgery. J Clin Med.

[2] Ostermann M, Bellomo R, Burdmann EA, Doi K, Endre ZH, Goldstein SL (2020). Controversies in acute kidney injury: conclusions from a Kidney Disease: Improving Global Outcomes (KDIGO) Conference. Kidney Int.

[3] Fortrie G, de Geus HRH, Betjes MGH (2019). The aftermath of acute kidney injury: a narrative review of long-term mortality and renal function. Crit Care.

[4] Benichel CR, Meneguin S (2020). Risk factors for acute renal injury in intensive clinical patients. Acta Paul Enferm.

[5] Vasco CF, Watanabe M, Fonseca CD, Vattimo MFF (2018). Sepsis-induced acute kidney injury: kidney protection effects by antioxidants. Rev Bras Enferm.

[6] Gonsalez SR, Cortês AL, Silva RC, Lowe J, Prieto MC, Silva Lara LD (2019). Acute kidney injury overview: from basic findings to new prevention and therapy strategies. Pharmacol Ther.

[7] Chen WY, Cai LH, Zhang ZH, Tao LL, Wen YC, Li ZB (2021). The timing of continuous renal replacement therapy initiation in sepsis-associated acute kidney injury in the intensive care unit: the CRTSAKI Study (Continuous RRT Timing in Sepsis-associated AKI in ICU): study protocol for a multicentre, randomised controlled trial. BMJ Open.

[8] Zhang J, Crichton S, Dixon A, Seylanova N, Peng ZY, Ostermann M (2019). Cumulative fluid accumulation is associated with the development of acute kidney injury and non-recovery of renal function: a retrospective analysis. Crit Care.

[9] French WB, Shah PR, Fatani YI, Rashid MM, Liebman ST, Cocchiola BJ (2022). Mortality and costs associated with acute kidney injury following major elective, non-cardiac surgery. J Clin Anesth.

[10] Cuschieri S (2019). The STROBE guidelines. Saudi J Anaesth.

[11] In J, Kang H, Kim JH, Kim TK, Ahn EJ, Lee DK (2020). Tips for troublesome sample-size calculation. Korean J Anesthesiol.

[12] Chawla LS, Bellomo R, Bihorac A, Goldstein SL, Siew ED, Bagshaw SM (2017). Acute kidney disease and renal recovery: consensus report of the Acute Disease Quality Initiative (ADQI) 16 Workgroup. Nat Rev Nephrol.

[13] Kuong-Guitton E, Buleje J (2023). The measurement of basal creatinine and the diagnosis of AKI with COVID-19. Int Urol Nephrol.

[14] World Health Organization (WHO) (2021). Draft recommendations for the prevention and management of obesity over the life course, including potential targets [Internet].

[15] Basak T, Aciksoz S, Tosun B, Akyuz A, Acikel C (2013). Comparison of three different thermometers in evaluating the body temperature of healthy young adult individuals. Int J Nurs Pract.

[16] Mehta RL (2020). Renal recovery after acute kidney injury and long-term outcomes: is time of the essence?. JAMA Netw Open.

[17] Gardner AK, Ghita GL, Wang Z, Ozrazgat-Baslanti T, Raymond SL, Mankowski RT (2019). The development of chronic critical illness determines physical function, quality of life, and long-term survival among early survivors of sepsis in surgical ICUs*. Crit Care Med.

[18] Realista S (2022). Acute kidney injury in the inpatient and outpatient setting. Crit Care Nurs Clin North Am.

[19] Abebe A, Kumela K, Belay M, Kebede B, Wobie Y (2021). Mortality and predictors of acute kidney injury in adults: a hospital-based prospective observational study. Sci Rep.

[20] Klinkhammer BM, Buchtler S, Djudjaj S, Bouteldja N, Palsson R, Edvardsson VO (2022). Current kidney function parameters overestimate kidney tissue repair in reversible experimental kidney disease. Kidney Int.

[21] Desai RJ, Kazarov CL, Wong A, Kane-Gill SL (2022). Kidney damage and stress biomarkers for early identification of drug-induced kidney injury: a systematic review. Drug Saf.

[22] Douvris A, Malhi G, Hiremath S, McIntyre L, Silver SA, Bagshaw SM (2018). Interventions to prevent hemodynamic instability during renal replacement therapy in critically ill patients: a systematic review. Crit Care.

[23] Zhou J, Zhang X, Lyu L, Ma X, Miao G, Chu H (2021). Modifiable risk factors of acute kidney injury after liver transplantation: a systematic review and meta-analysis. BMC Nephrol.

[24] Andonovic M, Shemilt R, Sim M, Traynor JP, Shaw M, Mark PB (2021). Timing of renal replacement therapy for patients with acute kidney injury: A systematic review and meta-analysis. J Intensive Care Soc.

[25] Pan HC, Chen YY, Tsai IJ, Shiao CC, Huang TM, Chan CK (2021). Accelerated versus standard initiation of renal replacement therapy for critically ill patients with acute kidney injury: a systematic review and meta-analysis of RCT studies. Crit Care.

[26] Tomašev N, Glorot X, Rae JW, Zielinski M, Askham H, Saraiva A (2019). A clinically applicable approach to continuous prediction of future acute kidney injury. Nature.

[27] Chandiraseharan V, Kalimuthu M, Prakash T, George T, Rajenesh A, Jayaseelan V (2020). Acute kidney injury is an independent predictor of in-hospital mortality in a general medical ward: A retrospective study from a tertiary care centre in south India. Indian J Med Res.

[28] Venditti EM, Wylie-Rosett J, Delahanty LM, Mele L, Hoskin MA, Edelstein SL (2019). Effect of wearable technology combined with a lifestyle intervention on long-term weight loss: the IDEA randomized clinical trial. JAMA.

[29] Milne B, Gilbey T, Ostermann M, Kunst G (2020). Pro: we should stop ace inhibitors early before cardiac surgery to prevent postoperative acute kidney injury. J Cardiothorac Vasc Anesth.

[30] Scholz H, Boivin FJ, Schmidt-Ott KM, Bachmann S, Eckardt KU, Scholl UI (2021). Kidney physiology and susceptibility to acute kidney injury: implications for renoprotection. Nat Rev Nephrol.

